# Live and carcass production traits for progeny of an F1 USDA Prime—Yield Grade 1 carcass clone sire compared to progeny of popular beef terminal sires

**DOI:** 10.1093/tas/txae126

**Published:** 2024-08-24

**Authors:** Forest L Francis, Becca B Grimes, Dean E Hawkins, David G Lust, Trent J McEvers, Travis C Tennant, Gregg O Veneklasen, Jason M Abraham, Justin F Gleghorn, Ty E Lawrence

**Affiliations:** Department of Agricultural Sciences, West Texas A&M University, Canyon, TX 79016, USA; Department of Agricultural Sciences, West Texas A&M University, Canyon, TX 79016, USA; Department of Agricultural Sciences, West Texas A&M University, Canyon, TX 79016, USA; Department of Agricultural Sciences, West Texas A&M University, Canyon, TX 79016, USA; Department of Agricultural Sciences, West Texas A&M University, Canyon, TX 79016, USA; Department of Agricultural Sciences, West Texas A&M University, Canyon, TX 79016, USA; Timber Creek Veterinary Hospital, Canyon, TX 79015, USA; Abraham Equine Inc., Canadian, TX 79014, USA; Cactus Feeders, Amarillo, TX79101, USA; Department of Agricultural Sciences, West Texas A&M University, Canyon, TX 79016, USA

**Keywords:** beef, carcass, cattle, clone, somatic cell nuclear transfer, terminal sire test

## Abstract

The cloning of beef carcasses that grade United States Department of Agriculture (USDA) Prime—yield grade (YG) 1 (P1) has produced a sire that ranked well against high-performing bulls from multiple breeds. An F1 (P1 × P1 - first generation offspring) sire would ideally outperform its high-performing parents. A terminal sire study was conducted comparing progeny of an F1 (P1 × P1) sire (AxG1) against progeny (heifers and steers) of four high-performing sires of varying breeds {P1 (ALPHA); Angus; Simmental; Angus × Simmental}. Production traits included morbidity and mortality frequencies, weaning weight, feedlot arrival weight, and days on feed; carcass traits included frequency of abscessed liver and lung health, quality grade and YG parameters, total carcass value (US$), and carcass value per hundredweight (CWT [45.4 kg]; US$). A completely randomized experimental design was used; data were analyzed using a mixed model with a fixed effect of sire and random effects of harvest date, sex, and pen. AxG1 sired heifers had the highest (*P* < 0.01) marbling score, the highest (*P* < 0.01) carcass value per CWT, and numerically had the lowest calculated YG and highest frequency of YG one carcass. Steers sired by AxG1 had the least (*P* = 0.05) backfat, lowest (*P* < 0.01) calculated YG, highest (*P* < 0.01) marbling score, highest (*P* < 0.01) frequency of USDA Prime carcasses, the greatest (*P* < 0.03) total carcass value, and greatest (*P* < 0.01) carcass value per CWT. Collectively, AxG1 steer and heifer carcasses exhibited the least 12th rib fat thickness and lowest USDA YG in addition to the largest longissimus muscle area, highest marbling score, and greatest frequency of USDA Prime. These data suggest that AxG1 performed comparably to other high-performing industry terminal sires in carcass quality and YG outcomes.

## Teaser

The progeny of an F1 USDA Prime—Yield Grade 1 carcass clone sire outperformed high-quality industry sires in quality and yield outcomes.

## Introduction

With the shift to a value-based marketing system, commercial cattle producers make breeding and management decisions based on what will maximize profitability for their operation. Commercial beef cattle production is generally most profitable when crossbreeding systems are utilized to capture heterosis and breed differences in future generations (Gregory and Cundiff, 1980; MacNeil, 2005).

The cloning project coined “PrimeOne”, has allowed our team at West Texas A&M University (WTAMU) to salvage rare and desirable genetics that producers in the commercial cattle industry seek. In 2012 the “PrimeOne” project cloned a steer carcass resulting in a bull (14% Zebu, 86% Angus; ALPHA) and a heifer carcass resulting in three heifers (GAMMA) produced via somatic cell nuclear transfer from carcasses that graded United States Department of Agriculture (USDA) Prime—yield grade (YG) 1 (P1); a rare and antagonistic outcome observed in only 0.07% of the U.S. beef population (Boykin et al., 2017). In a terminal sire study, live and carcass production traits for ALPHA progeny were compared to progeny of purebred Angus, Charolais, and Simmental reference sires (Sperber et al., 2023). For all outcomes that differed (*P* < 0.05), ALPHA progeny ranked either first or second; suggesting that ALPHA progeny performed comparably against other high-performing reference sires for terminal production traits (Sperber et al., 2023).

The subsequent breeding of ALPHA to GAMMA heifers produced 13 progeny in 2015 via embryo transfer (ET) into recipient cows. An F1 sire (AxG1) would ideally outperform its high-performing carcass quality and YG parents (ALPHA and GAMMA). Due to ALPHA’s progeny’s performance in a terminal sire study (Sperber et al., 2023), our hypothesis was that AxG1 would be a higher-performing terminal sire with progeny exhibiting live and carcass traits highly desired by commercial cattle producers. A terminal sire study was conducted to compare the progeny of four sires (purebred Angus, purebred Simmental, Angus × Simmental, and ALPHA) selected for their carcass and production traits to the progeny of AxG1 to determine his success in the terminal sire system.

## Materials and Methods

The cloning procedure that created ALPHA and the GAMMAs and the AI and ET procedures used to create AxG1 was completed under Institutional Animal Care and Use Committee (IACUC) protocol 031114. The progeny produced in this study were conceived from semen provided to Cactus Feeders (Amarillo, TX) to be used for artificial insemination (AI). All live cattle in the current study were under the direct care and supervision of Cactus Feeders and all live cattle data was collected by their employees. All experimental procedures followed the guidelines described in the Guide for the Care and Use of Agricultural Animals in Agricultural Research and Teaching (FASS, Savoy, IL).

### Sire Cloning Procedure

The sire that is the focus of this study, “AxG1”, is the progeny of two P1 carcass clones. The cloning process that resulted in the sire “ALPHA” and the dams “GAMMAs” was described by Sperber et al. (2023). ALPHA semen was utilized to inseminate GAMMA cow oocytes via AI and fertilized ova were transferred into recipient cows at the WTAMU Nance Ranch (Canyon, TX) via ET. The breeding resulted in nine bull and four heifers AxG progeny born in April 2015; seven males were castrated and two males remained intact (AxG1 and AxG2). We hypothesized that AxG steers would exhibit desirable live and carcass performance traits as calves until harvest. The AxG steers were placed into the WTAMU Feedlot (Canyon, TX) and fed for 182 days until the desired quality grade was reached for harvest. At harvest in May 2016 at the WTAMU Meat Lab (Canyon, TX), AxG steers exhibited an average USDA marbling score of Moderate 30 (Md30, USDA High Choice), average longissimus muscle area (LMA) of 96.9 cm^2^, and an average calculated USDA YG of 2.1. All listed AxG steer carcass metrics were highly desirable by beef producers, indicating that AxG steers performed well against industry standards. With such promising terminal outcomes from 13-mo-old steers, the hypothesis that AxG1 could be a high-performing terminal sire arose.

### Randomization and AI

British × Continental beef cows (*n* = 991) were bred via AI following estrus synchronization November 28-30, 2016. Cows were bred with semen from one of the five sires: ALPHA, AxG1, a purebred black Angus (Rampage; ANG), a purebred Simmental (Sure Bet; SIM), and an Angus × Simmental (Protégé; ANG-SIM). Straws of semen for each sire were thawed 10 at a time and dams were inseminated as they entered the chute randomly. On the last day of AI, semen for Rampage became unavailable and semen from Protégé was substituted. Total semen straw counts used for each sire were: 257-AxG1, 241-Alpha, 245-Sure Bet, 171-Rampage, and 77-Protégé.

Rampage, the Angus sire reported by ABS Global (De Forest, WI) as Quaker Hill Rampage 0A36 AAA 16925771 (ANG), was the Angus breeds’ top sire for registrations in 2018, was the breed leader for $Beef, $Feedlot, and $Weaning and was the top 1% of the Angus breed for REA and FAT. Sure Bet, the Simmental sire reported by ABS Global (De Forest, WI) as Dikemans Sure Bet ASA 2294262 (SIM), was ranked in the breed’s top 10% for marbling. Protégé, the Angus × Simmental sire reported by ABS Global (De Forest, WI) as TSN Protégé Z896 ASA 2699504 (ANG-SIM), was in the top 1% of its breed for weaning weight and hot carcass weight (HCW).

### Calf Identification and Weaning

Calves in this study (*n* = 739) were born in the fall of 2017 at Syracuse Feedyard (Cactus Feeders; Amarillo, TX), a confined cow-calf operation located east of Syracuse, KS. Gestation and parturition occurred in feedyard pens. At birth, calves received a visual tag that identified their birth date, Inforce 3 (Zoetis Inc., Kalamazoo, MI), and Calf Guard (Zoetis Inc.); bull calves were castrated with elastic band and administered tetanus vaccination. Calves that were treated for illness received an additional ear tag that identified the date and treatment. Health data for calves receiving treatment was recorded in the feedyards’ database system for later reporting.

At birth, Syracuse Feedyard employees evaluated dam maternal abilities and calf health. Calves born in poor health or to dams unfit to rear a calf (*n* = 30) were transported to Fullmer Calf Ranch located in Syracuse, KS, where they received enhanced care. Of the calves raised at Fullmer, the SIM and ANG-SIM each sired 1 calf, AxG1 sired 3 calves, ALPHA sired 5 calves, and ANG sired 6 calves.

Intermediate processing of calves (*n* = 709) occurred on November 17, 18, 19, 26, and 27, 2017. Calves received BoviShield GOLD 5 (Zoetis Inc.) and Ultrabac 7 (Zoetis Inc.), an additional visual identification tag, and an electronic identification tag. Ear tissue punches were taken for each calf and compared against semen from each sire by Quantum Genetix (Saskatoon, SK, CAN) for parentage testing. The calf count for each sire was: AxG1 (*n* = 105), ALPHA (*n* = 87), ANG (*n* = 77), SIM (*n* = 97), and ANG-SIM (*n* = 55). The remaining calves (*n* = 11) did not have sufficient DNA to allow for parentage testing.

On March 5, 7, and 8, 2018, calves in Syracuse Feedyard were weaned and calves from Fullmer were reintroduced into the feedyard population. During the weaning procedure, calves were implanted with Revalor-G (40 mg trenbolone acetate + 8 mg estradiol; Intervet Inc., Millsboro, DE), administered CyLence Pour-On Insecticide (Bayer Health Care LLC, Shawnee Mission, KS), Synanthic (Boehringer Ingelheim Vetmedica Inc., St. Joseph, MO), Dectomax (Zoetis Inc.), Presponse SQ (Boehringer Ingelheim Vetmedica Inc.), and Ultrabac 7 (Zoetis Inc.); weaning weights were recorded.

### Feedyard Arrival and Processing

On June 21, 2018 calves (*n* = 652) were sorted by sex and shipped from Syracuse Feedyard to Ulysses Feedyard (Cactus Feeders; Amarillo, TX) in Ulysses, KS. Upon arrival at Ulysses, steers (*n* = 363) were sorted five ways and heifers (*n* = 289) were sorted four ways for a total of nine finishing pens.

Sorting was achieved by utilizing a combined frame and weight measuring system (CCSS) from Performance Cattle Company (Amarillo, TX). The CCSS system was designed to classify cattle via algorithms utilizing frame dimensions and weight collected during arrival or intermediate processing. The system allows feedyards to select the number of sort pens desired and uniformly classifies cattle into pens while also estimating harvest dates.

During arrival sorting, steers were implanted (based on arrival weight) with either Revalor-IS (80 mg trenbolone acetate + 16 mg estradiol; Intervet Inc.; Pens 159, 160, and 161) or Revalor-XS (200 mg trenbolone acetate + 40 mg estradiol; Intervet Inc.; Pens 162 and 163). Heifers were implanted with either Revalor-IH (80 mg trenbolone acetate + 8 mg estradiol; Intervet Inc.; Pens 164 and 165) or Revalor-200 (200 mg trenbolone acetate + 20 mg estradiol; Intervet Inc.; Pens 166 and 167). All cattle were administered Bovishield 3 (Zoetis Inc.), Dectomax (Zoetis Inc.), Synanthic (Boehringer Ingelheim Vetmedica Inc.), and Nasalgen IP (Merck Inc., Summit, NJ). Heifers were rectally palpated via ultrasound to test for pregnancy. For heifers (*n* = 2) which were determined pregnant, Lutalyse (Zoetis Inc.) and dexamethasone injections were administered to initiate abortion. Males identified as bulls (*n* = 7) were castrated via emasculator.

For cattle in pens 159, 160, 161, 164, and 165 a terminal implant was not administered at initial processing and thus required a reimplant. On September 5, 2018 (77 d on feed), steers in pens 159, 160, and 161 were implanted with Revalor XS (200 mg trenbolone acetate + 40 mg estradiol; Intervet Inc.) and administered Bovishield 3 (Zoetis Inc.). On November 7, 2018 (140 d on feed), heifers in pen 164 were implanted with Revalor-200 (200 mg trenbolone acetate + 20 mg estradiol; Intervet Inc.) and administered Bovishield 3 (Zoetis Inc.). On November 12, 2018 (145 d on feed), heifers in pen 165 were implanted with Revalor-200 (200 mg trenbolone acetate + 40 mg estradiol; Intervet Inc.) and administered Bovishield 3 (Zoetis Inc.).

At arrival processing at Ulysses Feedyard, Syracuse Feedyard calf tags, and hospital tags were removed, and health data was reported for all animals. Each animal received a lot of identification tags with individual identification. Morbidity and mortality for animals were recorded via the feedyard lot tag’s individual identification number and reported in the feedyard’s health data system.

The diet that all cattle received was equivalent and included steam-flaked corn, corn stalks, wet distiller’s grain, liquid fat, urea, a vitamin and mineral supplement, and monensin sodium, tylosin phosphate, and ractopamine hydrochloride. Feed delivered was recorded daily and maintained electronically in the feedyard operating system. Days on feed (DOF) varied between pens due to sex and individual growth characteristics.

### Slaughter and Grading Procedures

Steers and heifers were slaughtered in their five-way (steers) or four-way (heifers) arrival sort pens beginning with heifers in pen 167 being harvested on November 12, 2018 (145 total DOF). Steers from pen 163 and heifers from pen 166 were harvested on November 26, 2018 (159 total DOF). Heifers from pen 165 were harvested on December 19, 2019 (182 total DOF) and steers from pen 162 were harvested on January 2, 2019 (196 total DOF). Steers from pen 161 were harvested on January 7, 2019 (201 total DOF). Heifers from pen 164 and steers from pen 160 were harvested on January 21, 2019 (215 total DOF). Steers from pen 159 were the final lot to reach harvest on February 20, 2019 (245 total DOF). All cattle were shipped to a commercial beef abattoir (USDA Est. # 278) located 90 km from Ulysses Feedyard.

All slaughter data was obtained by trained data collectors from West Texas A&M University-Beef Carcass Research Center (BCRC; Canyon, TX). Individual visual identification tags were manually recorded, electronic identification tags (EID) were recorded via Allflex RS420 Series Stick Reader (Allflex USA, DFW Airport, TX) and WTAMU identification tag was attached to carcasses via shroud pin. Liver and lung health outcomes were evaluated and recorded. Lungs were visually evaluated and manually palpated to determine the presence and severity of lung lesions, interlobular adhesions, plural adhesions, missing lobes, and interlobular fibrin tags. Lung scores were Normal (healthy lungs), Minor (presence of minor fibrin tags), Extensive (presence of extensive fibrin tags), 1 (5%-15% consolidated lung tissue), 2 (15%-50% consolidated lung tissue), and 3 (>50% consolidated lung tissue) following the procedures of Tennant et al. (2014). Livers were visually evaluated to determine health according to the scoring system reported by Brown and Lawrence (2010). Liver scores were Edible (no abnormalities), A- (1 or 2 small abscesses), A (1 or 2 large abscesses or multiple small abscesses), A + (multiple large abscesses), A + AD (liver was adhered to diaphragm or gastrointestinal tract), A + OP (ruptured liver abscess), A + AD/OP (ruptured abscess and adhered to diaphragm or gastrointestinal tract); other abnormalities recorded were cirrhosis, distoma, and telangiectasis (Brown and Lawrence, 2010). Following visceral evaluation, the packer identification tag number and HCW were recorded.

Following a 28 h chill, carcasses were ribbed for USDA-AMS grading. Cattle were quality and yield graded via the VBG2000 camera (E + V Technology, Oranienburg, Germany). Kidney, pelvic, and heart fat percentage for camera calculated YG was determined via a beef processor-specific algorithm. Empty body fat percentage (EBF; %) was estimated with an equation reported previously by Guiroy et al. (2002). Total carcass value (US$) and carcass value per hundredweight (CWT [45.4 kg]; US$) were calculated using a carcass base price of US$190.71/CWT derived from the U.S. Department of Agriculture Agricultural Marketing Service (AMSa, 2019), and carcass value pricing (Table 1) representative of the total duration of the project from calf conception date to last calf harvest date (November 2016 to February 2019).

**Table 1.** Carcass value-based pricing grid for duration of project^*^ (AMSb, 2019)

Base carcass price^*^ (AMSa, 2019)	Quality grade	Hot carcass weight, kg	Yield grade
		181-227 (−29.71)	1.0-2.0 (+3.75)
	Prime (+12.68)	227-250 (−21.34)	2.0-2.5 (+2.00)
	CAB (+3.74)	250-272 (−8.37)	2.5-3.0 (+1.63)
** **$190.71/CWT	Choice (0.00)	272-408 (0.00)	3.0-4.0 (0.00)
	Select (−11.39)	408-454 (−1.68)	4.0-5.0 (−11.50)
	Standard (−29.06)	454-476 (−7.18)	>5.0 (−17.19)
		>476 (−23.70)	

^*^Project duration was considered calf conception date to last calf harvest date (November 2016—February 2019). Provided as US$ per hundredweight (45.4 kg; CWT).

A power test (PROC POWER, α = 0.05; β = 0.90, SAS Institute, Cary, NC) was conducted to determine an appropriate number of strip loins per sire in each pen to collect for tenderness evaluations. Three Certified Angus Beef (CAB), USDA Institutional Meat Purchase Specifications 180 strip loins were collected from each sire within each pen for Warner-Bratzler shear force (WBSF) determinations. All sires in the study were genetically homozygous black; thus, the decision to collect CAB carcasses was made to reflect the quality standards associated with the brand. Strip loins were collected following the fabrication of carcasses and were vacuum-sealed and transported to the WTAMU meat lab (Canyon, TX). Due to the standards required for cattle to qualify for CAB and the distribution of sire progeny across pens, incomplete loin collections occurred in multiple sires and pens. Strip loin totals for sire and sex were: 12 AxG1 heifers, 12 Alpha heifers, 9 ANG heifers, 10 SIM heifers, 5 ANG-SIM heifers, 15 AxG1 steers, 15 Alpha steers, 14 ANG steers, 13 SIM steers, and 6 ANG-SIM steers.

### WBSF procedure

Collected strip loins were wet aged 14 days after harvest date at 3 °C; on day 14 loins were frozen at −29 °C for storage. Frozen strip loins were cut into steaks 2.54-cm-thick and the cranial end steak was vacuum sealed and kept frozen at −29 °C. Steaks were thawed at 5 °C for 24 h, cooked in a forced-air convection oven (Blodgett DFG-100-3, G.S. Blodgett Corporation, Essex Junction, VT) set at 177 °C to an internal temperature of 71 °C. The internal temperature was determined via Omega MDSSi8 meter (Omega Engineering Inc., Stamford, CT) and 0.001-gauge Omega Precision Fine Wire Thermocouples (Omega Engineering Inc., Stamford, CT). Cooked steaks were plastic wrapped and chilled for 24 h at 5 °C, then six cores (1.27 cm diameter) were removed parallel to the muscle fibers of each steak. Cores were sheared (Instron 5944, Instron, Norwood, MA) with a WBSF shear blade attachment at 250 mm/min. Shear blade specifications are reported in the American Meat Science Association Research Guidelines for Cookery, Sensory Evaluation, and Instrumental Tenderness Measurements of Meat (AMSA, 2016).

### Statistical Analysis

A completely randomized experimental design was utilized; individual animals were considered the experimental unit. Syracuse Feedyard heifer and steer data were jointly analyzed. For Ulysses Feedyard data and carcass data, heifers and steers were analyzed separately. Continuous and frequency data were analyzed using PROC MIXED and PROC GLIMMIX procedures of SAS 9.4 (SAS Institute Inc.), respectively. The fixed effect of sire was utilized and random effects of Syracuse pen, Ulysses pen, and harvest date were used. The Kenward-Roger’s approximation for denominator degrees of freedom was used to correct unequal cell sizes. Additionally, DOF (200 d), HCW (375 kg for heifers and 400 kg for steers), 12th rib subcutaneous backfat thickness (FAT; 1.3 cm), and marbling score (Modest^00^) were utilized as covariates in separate models. Least square means were generated with the LSMEANS option of SAS and means were separated and denoted differently (*P* ≤ 0.05) using the pairwise comparison PDIFF option of SAS (SAS Institute Inc.). Significance was determined at (*P* ≤ 0.05) and tendencies were observed at (0.05 < *P* ≤ 0.10).

## Results and Discussion

Summary statistics of the variation in measured heifer and steer progeny outcomes among the five sires (ALPHA, AxG1, ANG, SIM, ANG-SIM) utilized in this terminal sire test are reported in Table 2. The mean DOF for heifers was 167 d and ranged from 144 to 214 d; the mean DOF for heifers in this study falls within the range reported in the 2015 feedlot consulting nutritionist survey (150 to 350 d; Samuelson et al., 2016); however, the minimum DOF for heifers (144 d) was slightly less than the survey’s reported range. Additionally, the mean DOF for steers in this study was 195 d and ranged from 158 to 244 d with all values falling within the ranges reported in the 2015 feedlot consulting nutritionist survey (Samuelson et al., 2016). When comparing the mean carcass outcomes from the current study to the values reported in the 2016 National Beef Quality Audit (NBQA; Boykin et al., 2017), heifers had lower HCW (360 vs. 375 kg), less FAT (1.47 vs. 1.6 cm), and lower calculated YG (2.9 vs. 3.1) while exhibiting increased LMA (92.9 vs. 89.5 cm^2^) and greater marbling scores (531 vs. 477). Conversely, steers in the current study had heavier HCW (424 vs. 398 kg), more FAT (1.72 vs. 1.3 cm), larger LMA (92.6 vs. 88.9 cm^2^), greater calculated YG (3.7 vs. 3.1), and greater marbling scores (567 vs. 467) compared to the 2016 NBQA (Boykin et al., 2017).

**Table 2.** Summary of the outcome variation for heifer and steer progeny in a five sire terminal sire test

	Mean	Standard deviation	Minimum	Maximum
*Heifers*				
Days on feed^*^	167	24.4	144	214
Days of age at harvest	455	23.6	409	512
HCW, kg	360	26.9	293	435
Backfat, cm	1.47	0.33	0.67	2.49
LMA, cm^2^	92.90	7.09	74.80	115.02
Calculated yield grade^†^	2.86	0.63	1.02	4.71
Marbling score^‡^	531	92.5	355	837
Empty body fat^‖^, %	30.54	2.37	24.47	38.34
Carcass Value^$^, $/CWT	193.44	4.35	177.53	205.39
Carcass Value^¶^, $	1534.52	111.11	1246.83	1810.91
*Steers*				
Days on feed^*^	195	27.9	158	244
Days of age at harvest	481	26.5	434	541
HCW, kg	424	31.9	338.83	514.37
Backfat, cm	1.72	0.35	1.03	2.98
LMA, cm^2^	92.56	7.12	69.95	111.14
Calculated yield grade^†^	3.65	0.59	2.25	5.30
Marbling score^†^	567	102.6	371	849
Empty body fat^‖^, %	33.26	2.36	27.58	40.05
Carcass Value^$^, $/CWT	188.44	9.36	155.51	205.39
Carcass Value^¶^, $	1755.62	105.69	1441.47	2046.47

^*^Days on feed: calculated from date of Ulysses, KS, feedlot arrival to date of harvest.

^†^USDA yield grade equation: 2.60 + (0.98425 × 12^th^ rib fat, cm) + (0.2 × KPH, %) + (0.00837 × HCW, kg)—(0.0496 × LM area, cm^2^); USDA (2016).

^‡^Marbling score: 400 = small^00^, minimum required for U.S. Low Choice, 500 = modest^00^, minimum required for U.S. Premium Choice.

^‖^17.76207 + (4.68142 × 12th rib fat, cm) + (0.01945 × HCW, kg) + (0.81855 × quality grade; 4 = Select, 5 = Choice -, 6 = Choice, 7 = Choice +, 8 = Prime)—(0.06754 × longissimus muscle area, cm^2^); Guiroy et al. (2002).

^$^Provided as US$ per hundred weight (CWT; 45.4 kg).

^¶^Provided as US$.

Heifers in the current study have decreased HCW, FAT, and calculated YG compared to the most recent NBQA means is plausible considering the mean DOF for the study was in the low range reported in the 2015 feedlot consulting nutritionist survey (Samuleson et al., 2016) and HCW, FAT, and calculated YG are known to increase as feeding period DOF increase (Kirkpatrick et al., 2023). Conversely, to heifers, steers in the current study had increased HCW, FAT, and calculated YG compared to the 2016 NBQA means. The mean DOF in the 2015 feedlot nutritionist survey was 201 d which was only 6 d more than steers in the current study (195 d), however, the mean value from the survey included DOF values from feedlots with Holstein steers which would have increased DOF compared to native beef steers. Thus, it may be appropriate to compare the mode (180 DOF) of the survey data because it would represent a larger proportion of native cattle that were fed in the current study. Steers in the current study were on feed for 15 d greater than the mode from the 2015 feedlot consulting nutritionist survey (180 d; Samuelson et al., 2016), potentially explaining why our steers had increased HCW, LMA, and calculated YG compared to the 2016 NBQA (Boykin et al., 2017).

The increase in LMA and marbling score observed in the heifers and steers in our study compared to the 2016 NBQA means are likely due to the high-quality sire genetics used in the trial which were selected for being top-ranking terminal sires in their respective breeds.

### Gestation

Calves born from test sires in Syracuse Feedyard did not differ (*P* = 0.13) in mean gestation length (Table 3). The average gestation period length across all sires was 283 d, the general industry mean gestation length for beef cattle (Livesay and Bee, 1945). Cows bred to ALPHA averaged 284 d gestation period whereas calves from the remaining four sires averaged 282 d gestation period. Gestation length for AI-sired calves born at Syracuse Feedyard ranged from 268 to 328 d. Foote (1981) reported that the gestation length of beef cows exceeded 300 d at a frequency of 1.07%. Additionally, Sobek (2015) reported an extreme gestation length of 307 d in Simmental and Montbeliard breeds of cattle. Although unusual, a few cattle (*n* = 7) in this study exceeded the 307 d gestation period reported by Sobek (2015). A variety of factors including dam breed, sire breed, age, parity number, body condition score and weight of dam, seasonal effects, sex of calf, and nutrition status of the dam have all been reported to affect the gestation length of beef cattle (Andersen and Plum, 1965). Variations of dams utilized, the limit-fed diet offered to dams throughout gestation, and differences between sires could have all affected the wide range observed in gestation length.

**Table 3.** Gestation, calf performance, and health

Outcome	AxG1	Alpha	Angus	Simmental	Angus × Simmental	SEM	*P* -value
*N*	105	87	77	97	55	—	—
Gestation length, d	282	284	282	282	282	1.1	0.13
Female, %	43.8	41.4	44.2	43.3	47.3	—	—
Male, %	56.2	58.6	55.8	56.7	52.7	—	—
Weaning weight, kg	196.0^bc^	188.0^c^	210.7^a^	205.8^ab^	194.9^bc^	6.3	<0.01
Morbidity, %	24.8^a^	15.0^ab^	10.8^b^	9.2^b^	17.9^ab^	—	0.05

^a,b,c^Means within a row lacking a common superscript letter differ (*P *≤ 0.05).

### Weaning Weight

At weaning, a difference (*P* < 0.01) in weight (kg) was observed (Table 3); ANG-sired calves were heaviest (210.7 kg), followed by SIM (205.8 kg), AxG1 (196.0 kg), ANG-SIM (194.9 kg), and ALPHA sired calves exhibited the lowest average weaning weight (188.0 kg). Weaning weights across all sires averaged 201 kg and ranged from 58 to 297 kg. Average days of age at weaning across all sires was 180 d and ranged from 136 to 194 d. ANG and ANG-SIM’s expected progeny differences (ABS, 2019) report the sires to be in the top 1% of their respective breeds for weaning weight, thus higher weaning weights were not surprising outcomes for those sires.

In a profitable beef operation weaning of calves is accomplished to begin transitioning calves from reliance on their dams into stocker operations that will advance them through the beef production system. The average time of weaning for beef calves is around seven to eight months of age. However, a variety of factors affect the time of weaning including dam body weight, body condition score, suckling status, and nutrition status. Syracuse feedyard provided an additional dynamic to cow-calf production due to dams being limit-fed and all cattle being reared in confinement, thus the average age of weaning was lower than normal. Landaeta-Hernandez et al. (2013) reported that when fed in confinement, dams that were more dominant exhibited higher 90 d calf weight than dams that were subordinate. Dam body weight also followed the same trend as calf weight with dominant cattle obtaining a higher body weight than subordinate cattle (Landaeta-Hernández et al., 2013). Additionally, dams that are fed a low energy ration pre- and postpartum have been noted to have calves that exhibited lower weaning weights than dams fed a higher energy ration (Corah et al., 1975; Houghton et al., 1990). Due to the operations of the Syracuse feedyard, either of the previously mentioned reasons could have contributed to weaning weight variability across sires.

### Calf Morbidity

Calf morbidity differed (*P* = 0.05) between sires with AxG1 siring the highest percentage (24.8%) of treated calves with Alpha calves being intermediate to AxG1 and all other sires (Table 3). These results contrast with Sperber et al. (2023) in which Alpha sired calves were observed to have the lowest morbidity rate compared to all other sires. Overall morbidity reported by Sperber et al. (2023) was higher (27%) than reported in this study (15.3%); however, the total number of morbidities in Alpha-sired calves was similar between studies. Calves reared in confinement have been reported to have an increased frequency of morbidity for scours and bovine respiratory disease (BRD) than calves raised on pasture (Burson, 2017). Cattle raised in confinement are in much smaller enclosures whereby high levels of comingling are imminent; in conjunction with confined calves exhibiting a higher frequency of shedding bovine coronavirus and cryptosporidium (Burson, 2017), elevated calf morbidity was plausible. However, calves fed in confinement have exhibited elevated antibody titers to bovine viral diarrhea, bovine respiratory syncytial virus, infectious bovine rhinotracheitis, and parainfluenza 3; all viruses associated with BRD development (Burson, 2017). Thus, cattle fed in confinement may have a more enriched immune response than pasture-fed cattle and could be more prepared for comingling during the marketing process or during feedlot arrival. Mortality data was unable to be obtained in full due to incomplete records and was excluded from data analysis.

### Feedyard Performance

DOF was calculated as the number of days between the feedlot arrival date and harvest date and differed for both heifers and steers (*P* < 0.01) among sires (Table 4). Angus-sired calves exhibited the fewest DOF (*P *< 0.01) among all sires but did not differ from SIM and ANG-SIM for either steers or heifers. Sperber et al. (2023) also observed Angus-sired steer and heifer calves to require the least DOF among sires in their study. ALPHA-sired steers resulted in the most DOF (*P *< 0.01) among sires but did not differ from AxG1-sired steers. Similarly, ALPHA and AxG1-sired heifers had longer DOF (*P *< 0.01) but did not differ from one another. The notable increase in DOF-length for both ALPHA and AxG1 can be attributed to the later-maturing, *Bos indicus* influence as observed in a previous study (Sperber et al., 2023) in ALPHA-sired calves. Heifer DOF differed (*P* < 0.01) when FAT was included as a covariate in the model, but did not differ (*P* = 0.22) when the marbling score was used as a covariate, and tended to differ (*P* = 0.10) when HCW was used as a covariate. When using a constant 1.3 cm FAT in the model, outcomes mirrored the original no covariate model for both steers and heifers. Sperber et al. (2023) also observed an apparent decrease in DOF in British-type breeds utilized in their sire study when FAT was held constant. Steer DOF differed (*P* = 0.02) when HCW was used as a covariate and tended to differ (*P* = 0.06) when the marbling score was used as a covariate. When utilizing a constant HCW (400 kg), ALPHA-sired steers spent the longest DOF (208.3 d) and ANG-SIM-sired steers spent the least DOF (190.3 d), not differing from AxG1, ANG, and SIM-sired steers.

**Table 4.** Days on feed and days of age at harvest for heifers and steers from different sires utilized in a terminal sire test

		Days on feed^*^, d Covariate^†^ model				Days of age at harvest, d Covariate model			
	*N*	None	HCW	FAT	Marb	None	HCW	FAT	Marb
*Heifers*									
AxG1	46	174.5^a^	170.3	173.8^a^	165.8	461.8^a^	458.3	460.7^a^	452.3
Alpha	36	174.5^a^	168.7	173.8^a^	170.7	459.5^a^	454.6	458.5^a^	455.3
Angus	34	159.9^b^	160.1	158.8^b^	159.2	449.2^b^	449.2	447.6^b^	448.6
Simmental	42	160.8^b^	159.0	159.7^b^	160.7	448.4^b^	446.8	446.7^b^	448.1
Angus × Simmental	26	160.6^b^	158.2	159.9^b^	161.9	449.5^b^	447.4	448.6^b^	451.1
SEM		4.0	4.2	4.2	3.9	4.0	4.1	4.1	3.7
*P*-value		<0.01	0.10	<0.01	0.22	0.03	0.17	0.02	0.63
*Steers*									
AxG1	59	199.7^ab^	202.6^ab^	196.8^ab^	187.2	486.5^a^	488.8^ab^	484.2^a^	473.7
Alpha	51	206.3^a^	208.3^a^	202.6^a^	198.1	490.6^a^	492.3^a^	487.9^a^	482.5
Angus	43	188.4^c^	194.6^b^	184.5^c^	183.4	475.6^b^	480.6^bc^	472.7^b^	470.6
Simmental	55	189.8^bc^	192.7^b^	186.8^bc^	187.2	475.2^b^	477.6^c^	473.0^b^	472.9
Angus × Simmental	29	185.2^c^	190.3^b^	181.3^c^	182.8	472.7^b^	476.9^c^	469.9^b^	470.4
SEM		4.1	4.3	4.7	4.1	3.9	4.1	4.5	3.9
*P*-value		<0.01	0.02	<0.01	0.06	<0.01	0.02	<0.01	0.15

^*^Days on feed: calculated from date of Ulysses, KS, feedlot arrival to date of harvest.

^†^Covariate analysis model included constant values of 200 d on feed (DOF), hot carcass weight (HCW) of 375 and 400 kg for heifers and steers, 1.3 cm of backfat (FAT), and a marbling score (Marb) of Modest^00^.

^a,b,c^Means within a column within a sex lacking a common superscript letter differ (*P *≤ 0.05).

Heifer harvest age differed (*P* = 0.03) between sires; SIM (448.4 d), ANG (449.2 d), and ANG-SIM (449.5 d) had the youngest calves whereas Alpha (459.5 d) and AxG1 (461.8 d) had the oldest calves. Steer harvest age also differed (*P* < 0.01) between sires with ANG-SIM (472.7 d), SIM (475.2 d), and ANG (475.6 d) having the youngest calves and AxG1 (486.5 d) and ALPHA (490.6 d) having the oldest calves. Harvest age of heifers continued to differ (*P* = 0.02) when FAT was used as a covariate in a manner that mirrored the non-covariate model but did not differ when HCW (*P* = 0.17) or marbling score (*P* = 0.63) were covariates. The harvest age of steers did not differ (*P* = 0.15) when the marbling score was used as a covariate but differed (*P* < 0.02) when HCW and FAT were covariates. The oldest steers in the HCW covariate model were sired by Alpha (492.3 d) whereas SIM (477.6 d) and ANG-SIM (476.9 d) sired the youngest steers at harvest. Additionally, ANG (480.6 d) and AxG1 (488.8 d) sired similar aged steers; ANG steers differed from Alpha steers whereas AxG1 steers differed from SIM and ANG-SIM steers. Steer harvest age for the FAT covariate model differed in the same manner as the non-covariate model with Alpha and AxG1 sired steers differing from all other sires.

The utilization of the CCSS system from Performance Cattle Company (Garrison, 2005) sorted both heifers and steers based on body weight and animal body measurements into uniform harvest groups. It is likely that the greater DOF and older harvest age for AxG1 and ALPHA were due to a greater number of progeny from those sires that were sorted into later harvest groups by the CCSS system. Small-framed cattle will reach a final harvest size earlier than cattle of large frame size (Dolezal et al., 1993); early maturing cattle with smaller frame sizes would reach harvest at an earlier date and have fewer DOF than larger-framed cattle.

Morbidity during the finishing phase did not differ (*P* = 1.0) between sires for steers or heifers (Table 5). Steer progeny of ANG, ALPHA, and AxG1 had less than 2.5% morbidity whereas SIM and ANG-SIM had 0% morbidity. Heifers sired by AxG1 experienced 6.5% morbidity whereas no other heifers from any remaining sires had reported morbidity. Mortality did not differ (*P* = 1.0) between sires for steers or heifers. Heifers sired by SIM had a 5.1% mortality rate whereas no other sires had heifers with reported mortality. AxG1 steers had a 1.7% mortality rate whereas no other sires steers had reported mortality.

**Table 5.** Abscessed liver frequency and lung health for heifers and steers from different sires in a terminal sire test

		Morbidity, %	Mortality, %	Abscessed liver, %	Lung score^*^, %				
	*N*				1	2	3	M	E
*Heifers*									
AxG1	46	6.5	0	8.7	0	2.2	0	10.9	8.7
Alpha	36	0	0	2.9	5.9	0	0	8.8	14.7
Angus	34	0	0	8.8	5.9	8.8	5.9	23.5	11.8
Simmental	42	0	5.1	10.8	5.1	0	2.7	16.2	0
Angus × Simmental	26	0	0	15.0	6.7	5.0	0	40.0	15.0
*P*-value		1.0	1.0	0.68	0.92	0.81	0.98	0.09	0.92
*Steers*									
AxG1	59	1.7	1.7	7.0	1.7	3.5	1.8	1.8	21.1
Alpha	51	2.2	0	7.3	0	8.9	4.4	0	22.2
Angus	43	2.5	0	25.6	0	5.1	5.1	7.7	12.8
Simmental	55	0	0	14.8	1.9	9.3	3.7	0	14.8
Angus × Simmental	29	0	0	4.0	0	12.0	0	8.0	24.0
*P*-value		1.0	1.0	0.09	1.0	0.64	0.93	0.74	0.66

^*^Lung health: 1 = consolidation of lung lobes involving 5% to 15% of lung tissue; 2 = consolidation of lung lobes involving 15% to 50% of lung tissue; 3 = consolidation of lung lobes involving greater than 50% of lung tissue; M = minor fibrin tag formation on lung tissue; E = extensive fibrin tag formation on lung tissue; Tennant et al. (2014).

### Liver and Lung Health

No difference (*P* = 0.68) between sires was observed for the frequency of liver abscesses in heifers (Table 5); the frequency of liver abscesses by sire ranged from 2.9% to 15%. Liver abscesses in steers tended to differ (*P* = 0.09) across sires; ANG and SIM-sired steers had a 25.6% and 14.8% liver abscess rate, respectively, whereas ALPHA, AxG1, and ANG-SIM-sired steers all had less than 7.3% liver abscess rate. Liver abscesses have been a major economic liability to cattle producers and beef processors and were once ranked as the second highest concern of beef processors (Nagaraja and Chengappa, 1998). At slaughter, liver abscesses have been demonstrated to reduce carcass gain and dressing percentage in addition to lowering feed intake vs cattle with normal livers (Brinks et al., 1990). The notably low occurrence of liver abscesses in ALPHA-sired calves (5.1%) is a positive outcome for both producers and processors.

The frequency of heifers with lung consolidation scores 1, 2, 3, and extensive fibrin tag did not differ (*P* > 0.81) between sires (Table 5). Minor fibrin tag formation tended to differ (*P* = 0.09) between sires for heifers but extensive fibrin tags did not (*P* = 0.92). Heifers sired by ANG-SIM had the greatest frequency of minor fibrin tags at 40%, whereas AxG1 and ALPHA heifer progeny exhibited the lowest frequencies of minor fibrin tags, with 10.9% and 8.8%, respectively. There was no difference (*P* ≥ 0.64) between sires for frequencies of lung abnormalities in steers. Tennant et al. (2014) reported that advanced lung lesion scores had negative economic effects primarily associated with reduced HCW at slaughter. Overall lung health for the study was positive with a low frequency of advanced lung lesions for steers and heifers.

### Hot Carcass Weight

Least square means (Table 6) for heifer HCW (kg) differed (*P* < 0.01) between sires; ANG sired heifers resulted in the heaviest HCW (372.8 kg) whereas ALPHA sired heifers had the lowest (346.7 kg). Differences (*P* < 0.01) in HCW were also observed when DOF, FAT, and marbling scores were used as covariates in the model. Heifer HCW differed in the same manner for the non-covariate, DOF, and FAT models. In these models, Alpha sired the lightest heifers (341.5 to 346.7 kg) and ANG sired the heaviest heifers (366.5 to 372.8 kg). Additionally, AxG1, SIM, and ANG-SIM sired heifers with intermediate HCW and AxG1 did not differ from Alpha, whereas SIM and ANG-SIM did not differ from ANG. For the marbling score covariate model, Alpha sired heifers with the lightest HCW (344.3 kg) while ANG (371.2 kg) and SIM (361.3 kg) sired heifers with the heaviest HCW; AxG1 and ANG-SIM had intermediate HCW and AxG1 did not differ from Alpha, whereas ANG-SIM did not differ from ANG and SIM.

**Table 6.** Carcass outcomes including hot carcass weight (HCW), backfat, and longissimus muscle area (LMA) for steers and heifers from different sires in a terminal sire test

		HCW, kg Covariate^*^ model				Backfat, cm Covariate model				LMA, cm^2^ Covariate model				
	*N*	None	DOF	FAT	Marb	None	DOF	HCW	Marb	None	DOF	HCW	FAT	Marb
*Heifers*														
AxG1	46	352.9^bc^	347.8^bc^	349.0^bc^	347.8^bc^	1.45	1.47	1.56	1.32	93.8^a^	93.1^a^	94.7^a^	95.1^ab^	96.0^a^
Alpha	36	346.7^c^	341.5^c^	342.8^c^	344.3^c^	1.44	1.46	1.59	1.38	91.7^ab^	91.1^ab^	93.0^ab^	93.0^bc^	92.8^b^
Angus	34	372.8^a^	367.2^a^	366.5^a^	371.2^a^	1.52	1.55	1.53	1.50	93.0^a^	92.1^a^	93.0^ab^	94.9^ab^	93.2^ab^
Simmental	42	362.5^ab^	357.0^ab^	356.2^ab^	361.3^a^	1.52	1.55	1.58	1.50	94.6^a^	93.7^a^	95.0^a^	96.5^a^	95.1^ab^
Angus × Simmental	26	361.3^ab^	355.6^ab^	357.1^ab^	360.9^ab^	1.44	1.47	1.51	1.45	89.1^b^	88.2^b^	89.6^b^	90.3^c^	88.8^c^
SEM		6.3	7.4	6.0	6.9	0.06	0.08	0.06	0.06	1.3	1.6	1.4	1.2	1.2
*P*-value		<0.01	<0.01	<0.01	<0.01	0.67	0.64	0.87	0.08	0.05	0.05	0.05	<0.01	<0.01
*Steers*														
AxG1	59	420.0^bc^	420.6^bc^	412.9^ab^	416.7^bc^	1.64^b^	1.64^b^	1.58	1.52^b^	93.7	93.7	91.5^a^	95.6	95.4^a^
Alpha	51	414.0^c^	414.8^c^	404.2^b^	412.1^c^	1.76^a^	1.76^a^	1.73	1.69^a^	90.5	90.8	89.1^ab^	93.1	91.6^b^
Angus	43	434.2^a^	434.5^a^	424.1^a^	432.5^a^	1.78^a^	1.78^a^	1.69	1.72^a^	92.7	92.8	89.0^ab^	95.3	93.6^ab^
Simmental	55	415.2^c^	415.6^c^	407.5^b^	414.2^c^	1.67^ab^	1.67^ab^	1.63	1.64^ab^	92.5	92.4	90.8^a^	94.5	93.0^ab^
Angus × Simmental	29	430.3^ab^	430.6^ab^	419.5^a^	429.2^ab^	1.82^a^	1.82^a^	1.73	1.78^a^	90.6	90.4	87.3^b^	93.4	91.1^b^
SEM		8.3	8.1	7.8	8.6	0.08	0.09	0.08	0.08	1.3	1.1	1.2	1.5	1.3
*P*-value		<0.01	<0.01	<0.01	<0.01	0.05	0.05	0.11	<0.01	0.14	0.17	0.03	0.33	0.04

^*^Covariate analysis model included constant values of 200 d on feed (DOF), hot carcass weight (HCW) of 375 and 400 kg for heifers and steers, 1.3 cm of backfat (FAT), and a marbling score (Marb) of Modest^00^.

^a,b,c^Means within a column within a sex lacking a common superscript letter differ (*P *≤ 0.05).

Steer least square means for HCW differed (*P* < 0.01) between sires with ANG sired steers having the heaviest HCW (434.2 kg) and ALPHA and SIM steers having the lightest HCW (414.0 and 415.2 kg, respectively). Differences (*P* < 0.01) in HCW were also observed when DOF, FAT, and marbling scores were used as covariates. The non-covariate, DOF, and marbling score models all differed in the same way where Alpha and SIM steers had the lightest HCW whereas ANG steers had the heaviest HCW. In these models, AxG1 and ANG-SIM were intermediate and did not differ from each other, but AxG1 differed from ANG whereas SIM-ANG differed from Alpha and SIM. When FAT was a covariate in the model, Alpha and SIM had the lightest HCW whereas ANG and SIM-ANG had the heaviest HCW with AxG1 being intermediate and not differing from all other sires. HCW has been reported to account for 50%-92% of the total revenue of beef carcasses marketed on value-based grids (Tatum et al., 2006). Thus, heavier HCWs are typically sought after by beef producers.

### Fat Thickness

The least-square means (Table 6) for FAT (cm) of heifer progeny did not differ (*P* = 0.67) between sires. Similarly, for heifers, when DOF and HCW were used as covariates, FAT did not differ (*P* ≥ 0.64) between sires. However, when a constant marbling score of Modest^00^ was the covariate, FAT tended to differ (*P* = 0.08) between sires and ranged from 1.32 to 1.50 cm between the 5 sires. For steers, the least square means for FAT differed (*P* = 0.05) between sires; AxG1 sired calves were trimmer (1.64 cm) than calves from all other sires except for SIM (1.67 cm). When DOF and marbling score were used as covariates, FAT differed (*P* ≤ 0.05) between sires whereas when HCW was the covariate FAT did not differ (*P* = 0.11) between sires. In both the DOF and marbling score covariate models, AxG1 steers exhibited the least amount of FAT (1.52 to 1.64 cm) and differed from Alpha (1.69 to 1.76 cm), ANG (1.72 to 1.78 cm), and SIM-ANG (1.78 to 1.82 cm) whereas SIM sired steers (1.64 to 1.67 cm) did not differ from any other sires. Backfat thickness is the YG component most closely related to cutability and is an important metric for calculating the YG of beef carcasses (Lawrence, 2017). Fat thickness has been considered the most important variable in multiple regression equations designed to predict the percentage of boneless closely trimmed retail cuts of beef (Abraham et al., 1968).

#### Longissimus muscle area.

LMA (cm^2^) differed (*P* = 0.05) among sires for heifer progeny; SIM heifers (94.6 cm^2^), AxG1 heifers (93.8 cm^2^), and ANG heifers (93.0 cm^2^) had LMA larger than ANG-SIM heifers (89.1 cm^2^). Heifer LMA differed (*P* ≤ 0.05) when DOF, HCW, FAT, and marbling scores were used as covariates. Across all covariate models, ANG-SIM sired heifers had the smallest LMA (88.2 to 90.3 cm^2^). In the DOF, HCW, and FAT covariate models, SIM-sired heifers had the largest LMA (93.7 to 96.5 cm^2^), and in the marbling score covariate model, AxG1 heifers had the largest LMA (96.0 cm^2^). All other sires across models had intermediate LMA sizes and dependent on the statistical model differed from the largest or smallest LMA sire. No difference (*P *= 0.14) was observed for steer LMA among sires. Steer LMA did not differ between sires when DOF (*P* = 0.17) or FAT (*P* = 0.33) were covariates. However, differences (*P* ≤ 0.04) in LMA were observed when HCW and marbling scores were covariates. Steers sired by AxG1 had the largest LMA (91.5 to 95.4 cm^2^) for statistical models using HCW or marbling scores as covariates. Another important factor of the YG equation, LMA, has a moderate positive correlation coefficient (0.47) to cutability and can be a useful tool in creating cutability prediction equations (Crouse et al., 1975).

### Calculated YG

The least-square means for heifer (Table 7) calculated YG did not differ (*P* = 0.22) between sires. Heifers sired by AxG1 numerically had the lowest average YG of all sires (YG 2.73) when no covariate was used in the model. Calculated YG did not differ when DOF (*P* = 0.21) or HCW (*P* = 0.67) were used as covariates. However, differences (*P* < 0.01) in calculated YG were observed when FAT and marbling scores were covariates. In the FAT covariate model, SIM and AxG1 heifers had the lowest calculated YG (2.48 and 2.49, respectively), whereas ANG-SIM heifers had the highest calculated YG (2.80). This indicates at a constant FAT (1.3 cm) that SIM and AxG1 heifers had a larger LMA to HCW ratio than ANG-SIM heifers. Alpha and ANG heifers had intermediate YG in the FAT covariate model and did not differ from each other. Notably, when the marbling score was the covariate, AxG1 heifers had the lowest calculated YG (2.45) and ANG-SIM heifers had the highest calculated YG (3.04). The frequency of heifers stamped USDA YG 1, 3, 4, and 5 (Table 8) did not differ (*P* > 0.25) between sires. The frequency of heifers to grade YG 2 tended to differ (*P* = 0.08) between sires; Alpha-sired heifers were 73.6% YG 2 whereas ANG-SIM heifers were 30.0% YG 2. Although there was no difference (*P* > 0.25) for the frequency of heifers grading YG 1, 3, 4, and 5, it is noteworthy that AxG1 heifers graded 15.2% YG 1 and had 0% YG 4 carcasses. Notably, Alpha-sired heifers exhibited the highest percentage of YG 1 and 2 carcasses (76.5%). There were no YG 5 carcasses for any sires. Although backfat thickness for heifers was very similar across all sires, AxG1 heifers likely had the lowest average YG due to lighter HCW and larger LMA; thus, lowering carcass YG.

**Table 7.** Carcass outcomes including USDA calculated yield grade, marbling score, and empty body fat percentage for heifers and steers from different sires in a terminal sire test

			Calculated yield grade^*^ Covariate^†^ model						Marbling score^‡^ Covariate model						Empty body fat^‖^, %, Covariate model
	*N*	None	DOF	HCW	FAT	Marb	None	DOF	HCW	FAT	None	DOF	HCW	FAT	Marb
*Heifers*															
AxG1	46	2.73	2.74	2.98	2.49^c^	2.45^c^	583^a^	619^a^	599^a^	569^a^	30.7	31.0	31.7	29.7	29.1^c^
Alpha	36	2.77	2.78	3.09	2.54^bc^	2.64^bc^	539^b^	574^b^	559^b^	526^b^	30.3	30.6	31.6	29.4	29.6^bc^
Angus	34	3.02	3.03	3.03	2.65^ab^	2.96^a^	524^b^	562^b^	527^b^	502^b^	31.0	31.4	31.1	29.5	30.6^a^
Simmental	42	2.86	2.86	2.98	2.48^c^	2.79^ab^	521^b^	559^b^	531^b^	499^b^	30.6	31.0	31.2	29.1	30.3^ab^
Angus × Simmental	26	3.04	3.05	3.17	2.80^a^	3.04^a^	505^b^	542^b^	516^b^	490^b^	30.5	30.9	31.1	29.5	30.5^ab^
SEM		0.13	0.17	0.11	0.07	0.12	25.3	18.8	26.9	23.4	0.5	0.7	0.5	0.2	0.4
*P*-value		0.22	0.21	0.67	<0.01	<0.01	<0.01	<0.01	<0.01	<0.01	0.83	0.80	0.73	0.07	<0.01
*Steers*															
AxG1	59	3.48^c^	3.48^c^	3.37^c^	3.00	3.25^c^	630^a^	627^a^	625^a^	605^a^	33.3	33.2	32.7	31.2^a^	31.4^c^
Alpha	51	3.71^ab^	3.71^ab^	3.63^ab^	3.04	3.58^ab^	576^b^	574^b^	573^b^	541^b^	33.5	33.5	33.1	30.7^bc^	32.4^ab^
Angus	43	3.80^a^	3.80^a^	3.60^ab^	3.10	3.69^a^	562^bc^	563^b^	553^bc^	525^b^	33.7	33.7	32.8	30.8^b^	32.9^a^
Simmental	55	3.54^bc^	3.54^bc^	3.45^bc^	3.00	3.48^b^	539^c^	539^b^	535^c^	511^b^	32.7	32.7	32.2	30.4^c^	32.1^bc^
Angus × Simmental	29	3.91^a^	3.91^a^	3.73^a^	3.16	3.83^a^	543^bc^	542^b^	536^bc^	504^b^	33.8	33.8	33.0	30.6^bc^	33.2^a^
SEM		0.13	0.14	0.11	0.05	0.13	22.3	15.4	23.4	22.7	0.6	0.6	0.5	0.2	0.5
*P*-value		<0.01	<0.01	0.02	0.10	<0.01	<0.01	<0.01	<0.01	<0.01	0.09	0.09	0.28	<0.01	<0.01

^*^USDA yield grade equation: 2.60 + (0.98425 × 12th rib fat, cm) + (0.2 × KPH, %) + (0.00837 × HCW, kg)—(0.0496 × LM area, cm^2^); USDA (2016).

^†^Covariate analysis model included constant values of 200 d on feed (DOF), hot carcass weight (HCW) of 375 and 400 kg for heifers and steers, 1.3 cm of backfat (FAT), and a marbling score (Marb) of Modest^00^.

^‡^Marbling score: 400 = small^00^, minimum required for U.S. Low Choice, 500 = modest^00^, minimum required for U.S. Premium Choice.

^‖^17.76207 + (4.68142 × 12^th^ rib fat, cm) + (0.01945 × HCW, kg) + (0.81855 × quality grade; 4 = Select, 5 = Choice -, 6 = Choice, 7 = Choice +, 8 = Prime)—(0.06754 × longissimus muscle area, cm^2^); Guiroy et al. (2002).

^a,b,c^Means within a column within a sex lacking a common superscript letter differ (*P *≤ 0.05).

**Table 8.** Frequency of USDA carcass quality and yield grade outcomes of heifers and steers from different sires in a terminal sire test

		Quality grade, %				Yield grade, %				
	*N*	Prime	CAB^*^	Choice	Select	1	2	3	4	5
*Heifers*										
AxG1	46	10.8	60.9	26.1	2.2	15.2	47.8	37.0	0.0	0.0
Alpha	36	8.8	50.0	41.2	0.0	2.9	73.6	20.6	2.9	0.0
Angus	34	2.9	35.3	47.1	14.7	5.9	44.1	44.1	5.9	0.0
Simmental	42	0.0	48.7	51.3	0.0	5.4	51.4	40.5	2.7	0.0
Angus × Simmental	26	5.0	30.0	55.0	10.0	10.0	30.0	50.0	10.0	0
*P*-value		0.76	0.16	0.16	0.52	0.39	0.08	0.25	0.79	1.0
*Steers*										
AxG1	59	33.9^a^	48.2	17.9	0	0	16.1	67.9	16.0	0
Alpha	51	13.3^b^	57.8	28.9	0	0	8.9	62.2	26.7	2.2
Angus	43	5.1^b^	48.7	46.2	0	0	5.1	59.0	33.3	2.6
Simmental	55	3.7^b^	57.4	37.0	1.9	0	20.4	57.4	22.2	0
Angus × Simmental	29	4.0^b^	44.0	44.0	8.0	0	4.0	52.0	44.0	0
*P*-value		<0.01	0.68	0.07	0.83	1.0	0.18	0.68	0.13	1.0

^*^CAB: Certified Angus Beef; brand in which subprimals and retail cuts are marketed; carcasses meeting 10 quality standards as outlined and determined by the brand.

^a,b,c^Means within a column within a sex lacking common superscript letter differ (*P *≤ 0.05).

Calculated YG differed (*P* < 0.01) between sires for steer progeny (Table 7); AxG1 steers had the lowest average yield grade (YG 3.48) and differed from ANG (YG 3.80), ANG-SIM (YG 3.91), and ALPHA (YG 3.71) sired steers in the no covariate model. Calculated YG of steers tended to differ (*P* = 0.10) between sires when FAT was used as a covariate, with AxG1 and SIM having the lowest yield grade (YG 3.00). When DOF, HCW, and marbling scores were included as covariates, calculated YG differed (*P* ≤ 0.02) between sires with AxG1 steers exhibiting the lowest values (YG 3.25 to 3.48). The frequency of steers stamped YG 1-5 did not differ (*P* > 0.13) between sires. AxG1 steers numerically had the lowest frequency of YG 4 carcasses (16.0%) while also having the highest percentage of carcasses grading YG 2 and 3 (84%). There were no steers achieving YG 1 and Alpha and ANG were the only sires to have steers grading YG 5 (2.2% and 2.6%, respectively). AxG1 steers likely exhibited lower average YG due to having large LMA, moderate HCW, and low FAT; thus, lowering carcass YG.

Lawrence et al. (2008) reported that there was a linear relationship established that requires a minimum LMA per unit of HCW in calculating the YG equation. As HCW increases, the required LMA also increases; a carcass with a lighter HCW would require a smaller LMA to meet this relationship. However, if a carcass with a lighter HCW has a larger LMA than required, the YG of the carcass will numerically decrease. This is likely the case for AxG1 sired carcasses. Although the HCW for AxG1 sired steers and heifers were numerically lighter throughout the study, a larger LMA associated with these carcasses was sufficient to lower the YG.

### Quality Grading and Marbling Scores

The least-square means for heifer marbling scores (Table 7) differed (*P* < 0.01) between sires across all statistical models. AxG1 sired heifers had the highest marbling scores (569-619; Modest^69^–Moderate^19^) and differed from all other sires in all statistical models. The frequency for heifer carcasses to be stamped USDA Prime, G-1 (CAB), Choice, and Select (Table 8) did not differ (*P* > 0.16) between sires.

The least-square means for steer marbling scores (Table 7) differed (*P* < 0.01) between sires across all statistical models. AxG1 steers had the highest average marbling score (605-630; Moderate^05^–Moderate^30^) and differed from steers of all other sires in all statistical models. The frequency of steers (Table 8) to be stamped USDA G-1 (*P* = 0.68) and Select (*P* = 0.83) did not differ between sires. The frequency of steers grading USDA Choice tended to differ (*P* = 0.07) between sires; AxG1 steers had the lowest frequency of grading USDA Choice (17.9%) whereas ANG steers had the highest frequency (46.2%). The frequency of steers grading USDA Prime differed (*P* < 0.01) between sires; AxG1 steers had the highest frequency (33.9%) of USDA Prime stamped carcasses, differing from steers of all other sires. ALPHA-sired steers had the second highest frequency (13.3%) of USDA Prime stamped carcasses, a very notable figure, however, overshadowed by AxG1 grading performance.

Boykin et al. (2017) reported that the mean marbling score for the 2016 NBQA was 470 (Small^70^); though all sires in this study had numerically higher marbling scores than reported in the 2016 NBQA, AxG1 progeny had numerically over a 100-degree higher marbling score (610; Moderate^10^) than the audit average. Additionally, in the 2016 NBQA, 3.98% of carcasses graded USDA Prime (Boykin et al., 2017) whereas AxG1 progeny in this study graded 23.53% USDA Prime, approximately 6-fold greater than the frequency from the 2016 audit. AxG1 progeny were also more likely to grade USDA Prime than USDA Low or Commodity Choice (21.57%).

### Empty Body Fat (%)

The least-square means of heifer calculated EBF did not differ (*P *= 0.83) among sires (Table 7). Heifer EBF did not differ when DOF (*P* = 0.80) or HCW (*P* = 0.73) were used as covariates yet tended to differ (*P* = 0.07) when FAT was the covariate. However, EBF differed (*P* < 0.01) in heifers when the marbling score was the covariate; AxG1 heifers had the lowest calculated EBF (29.1%) and ANG heifers had the highest calculated EBF (30.6%). Steer EBF tended to differ (*P* = 0.09) between sires with ANG-SIM steers exhibiting the highest numeric EBF (33.8%) and SIM steers the lowest (32.7%). Steer EBF did not differ (*P* = 0.28) when HCW was the covariate yet tended to differ (*P* = 0.09) when DOF was the covariate. Additionally, steer EBF differed (*P* < 0.01) between sires when FAT and marbling score were covariates; AxG1 steers had the greatest EBF (31.1%) when FAT was the covariate and ANG-SIM or ANG steers had the greatest EBF (33.2% or 32.9%, respectively) when marbling score was the covariate. Guiroy et al., (2002) reported that as quality grade increased, EBF generally increased as well; USDA Choice cattle generally have lower EBF than USDA Prime cattle. The USDA Prime and YG 1 carcass that ALPHA was cloned from had a calculated EBF of 30.1%, lower than the average reported for USDA Prime carcasses by Guiroy et al. (2002). Steers sired by AxG1 were the highest-grading carcasses and had the lowest calculated EBF when the marbling score of Modest^00^ was used as a covariate. This can be attributed to AxG1 steer carcasses having the trimmest backfat thickness, second largest LMA, and second smallest HCW’s which would lower the carcasses EBF, even as carcasses graded better.

### Carcass Value

The least-square means for heifer carcass value per CWT differed (*P* < 0.01) between sires across all statistical models (Table 9). Heifers sired by AxG1 had the highest value per CWT ($193.80/CWT to $195.97/CWT) in all statistical models. Additionally, the total carcass value of heifers differed (*P* ≤ 0.05) between sires across all statistical models. Heifers sired by ANG had the greatest total carcass value ($1,547-$1,572) for the no covariate, DOF, FAT, and marbling score models due to ANG heifers having the highest HCW. When HCW is held constant in the statistical model, AxG1 heifers had the highest total carcass value ($1612) while ANG heifers had the lowest value ($1579). In all statistical models except when HCW was a covariate, ANG heifers had the highest total carcass value and the lowest value per CWT. The inverse is true for AxG1 and ALPHA heifers. This inverse relationship is due to AxG1 and ALPHA sired heifers having numerically the lowest HCW’s while ANG heifers had the highest HCW; ANG heifers had more pounds of carcass to sell even though their price per CWT was lower than AxG1 and ALPHA heifers.

**Table 9.** Total carcass value and carcass value per hundredweight (CWT) of heifers and steers from different sires utilized in a terminal sire test

		Total carcass value^†^, $ Covariate^*^ model					Carcass value^†^, $/CWT Covariate model				
	N	None	DOF	HCW	FAT	Marb	None	DOF	HCW	FAT	Marb
*Heifers*											
AxG1	46	1,521^bc^	1,502^bc^	1,612^a^	1,505^ab^	1,483^b^	195.48^a^	195.97^a^	195.12^a^	195.58^a^	193.80^a^
Alpha	36	1,487^c^	1,469^c^	1,604^ab^	1,472^b^	1,470^b^	194.72^ab^	195.21^ab^	194.24^ab^	194.81^ab^	193.56^a^
Angus	34	1,572^a^	1,551^a^	1,579^d^	1,547^a^	1,560^a^	191.09^d^	191.70^d^	191.04^c^	191.26^c^	190.74^c^
Simmental	42	1,546^ab^	1,526^ab^	1,596^bc^	1,521^a^	1,536^a^	193.39^bc^	193.99^bc^	193.16^b^	193.55^b^	193.02^ab^
Angus × Simmental	26	1,525^abc^	1,504^abc^	1,580^cd^	1,509^ab^	1,522^ab^	191.25^cd^	191.86^cd^	191.00^c^	191.36^c^	191.34^bc^
SEM		25.4	31.2	6.30	24.0	29.8	0.76	0.89	0.78	0.78	0.74
*P*-value		0.02	0.03	<0.01	0.05	<0.01	<0.01	<0.01	<0.01	<0.01	<0.01
*Steers*											
AxG1	59	1,788^a^	1,788^a^	1,733^a^	1,783^a^	1,754	193.22^a^	193.08^a^	196.21^a^	196.08^a^	190.93^a^
Alpha	51	1,730^b^	1,732^b^	1,693^b^	1,722^b^	1,711	189.85^b^	189.66^b^	191.97^b^	193.80^ab^	188.52^a^
Angus	43	1,764^ab^	1,762^ab^	1,667^bc^	1,757^ab^	1,747	184.52^c^	184.44^c^	189.48^bc^	188.55^c^	183.42^b^
Simmental	55	1,739^b^	1,738^b^	1,695^b^	1,734^b^	1,729	190.01^b^	189.93^b^	192.18^b^	193.13^b^	189.32^a^
Angus × Simmental	29	1,730^b^	1,728^b^	1,645^c^	1,723^b^	1,719	182.85^c^	182.78^c^	187.32^c^	187.19^c^	182.07^b^
SEM		18.2	18.0	13.4	20.3	21.0	2.24	2.26	1.34	1.96	2.24
*P*-value		0.03	0.03	<0.01	0.02	0.22	<0.01	<0.01	<0.01	<0.01	<0.01

^*^Covariate analysis model included constant values of 200 d on feed (DOF), hot carcass weight (HCW) of 375 and 400 kg for heifers and steers, 1.3 cm of backfat (BF), and a marbling score (Marb) of Modest^00^.

^†^Provided in US$.

^a,b,c^Means within a column within a sex lacking common superscript letter differ (*P *≤ 0.05).

Steer carcass value per CWT (Table 9) differed (*P* < 0.01) between sires across all statistical models. AxG1 steers had the highest value per CWT ($190.93/CWT-$196.21/CWT) and differed from all other sires in all statistical models except when FAT and marbling scores were covariates. No difference (*P* = 0.22) was observed for the total carcass value between sires in the marbling score covariate model. However, total carcass value differed (*P* ≤ 0.03) between sires in the no covariate, DOF, HCW, and FAT models; AxG1 steers had the highest total carcass value ($1,733-$1,788) across these statistical models. Unlike the occurrence in heifers, AxG1 steers had moderate HCW; this in addition to higher average USDA quality grades and lower average USDA YGs allowed AxG1 sired steers to have the highest carcass value per CWT and total carcass value.

### Warner-Bratzler Shear Force

The WBSF method of mechanical tenderness testing was developed in the 1920s by K.F. Warner (1952) and refined in 1932 at Kansas State University as a master’s degree thesis project for L.J. Bratzler (1932). Since its development, WBSF has become the gold standard for objective tenderness testing of meat.

The shear force value among sires (Table 10) for both heifer and steer progeny did not differ (*P* > 0.22). ASTM International (West Conshohocken, PA, USA) created a maximum tenderness threshold value (MTTV) of 4.4 kg in WBSF testing to be considered “Certified Tender” beef (ASTM, 2011). Additionally, the claim “Certified Very Tender” can be attained by obtaining an MTTV of less than 3.9 kg in WBSF testing. Both steers and heifers of all sires had mean WBSF values under the “Certified Very Tender” threshold. Frequencies of both steers and heifers to be “Certified Tender” and “Certified Very Tender” did not differ (*P* ≥ 0.62) between sires. Numerically, ANG-SIM sired heifers had the lowest frequency of “Certified Tender” and “Certified Very Tender” steaks (60.0% and 21.9%, respectively) whereas SIM sired heifers had the highest frequency (100% and 80%, respectively); the SIM sire was ranked in the top 1% of his breed for tenderness.

**Table 10.** Warner-Bratzler Shear Force outcomes of loin samples from heifer and steer carcasses for comparison in a terminal sire test

	*N*	Mean shear, kg	Tender^*^, %	Very tender^*^, %
*Heifers*				
AxG1	12	3.80	75.0	58.3
Alpha	12	3.48	91.7	66.7
Angus	9	3.59	88.9	66.7
Simmental	10	3.24	100.0	80.0
Angus × Simmental	5	3.40	60.0	21.9
SEM		0.25	—	—
*P*-value		0.22	0.62	0.87
*Steers*				
AxG1	15	3.4	100.0	100.0
Alpha	15	3.4	100.0	100.0
Angus	14	3.6	92.9	71.4
Simmental	13	3.5	100.0	76.9
Angus × Simmental	6	3.5	100.0	83.3
SEM		0.16	—	—
*P*-value		0.69	1.00	0.99

^*^Marketing claims certified by ASTM International and USDA AMS;.

Certified Tender ≤ 4.4 kg and Certified Very Tender ≤ 3.9 kg.

## Conclusion

Data from this project investigated the terminal sire traits for steers and heifers sired by an F1 USDA Prime—YG 1 carcass clone sire. Findings from this study suggest that the F1 bull, AxG1, outperformed his cloned sire, ALPHA, and three other high-power industry terminal sires of varying breeds in carcass quality and YG outcomes. The progeny of AxG1 exhibited carcass performance that would be highly desired by commercial cattle producers, validating that AxG1 was a high-performing terminal sire in the beef industry.
